# A Pilot Randomized Controlled Trial of a Single-Session Digital Acceptance and Commitment Therapy Intervention

**DOI:** 10.3390/bs15010075

**Published:** 2025-01-16

**Authors:** Michael E. Levin, Miriam N. Mukasa, Emily M. Bowers, Korena S. Klimczak, Ty B. Aller

**Affiliations:** 1 Department of Psychology, Utah State University, 2810 Old Main Hill, Logan, UT 84322, USA; 2 Institute for Disability Research, Policy, and Practice, Utah State University, Logan, UT 84322, USA

**Keywords:** acceptance and commitment therapy, eHealth, psychological flexibility, university students, single-session intervention

## Abstract

Adherence challenges are common among digital mental health interventions (DMHIs). Single-session DMHIs may help by providing a low-intensity intervention that takes less time to complete. This pilot randomized controlled trial sought to evaluate the acceptability and preliminary efficacy of a single-session DMHI based on acceptance and commitment therapy (ACT) in a non-clinical sample of college students. The trial was pre-registered (NCT06139718). A total of 61 students were recruited, which was below the target of 100 participants. The participants were randomized to the single-session ACT DMHI or to a waitlist condition, with assessments completed at 1-week and 1-month follow-ups. The vast majority of participants (87%) completed the single-session ACT program. The participants provided high program satisfaction ratings. There were no differences between conditions on psychological flexibility, distress, or mental health help seeking. However, the participants assigned to ACT improved significantly more than the waitlist on well-being from baseline to 1-month follow-up (*d* = 0.29). Overall, the results were mixed, with some support for the acceptability and potential efficacy of a single-session ACT DMHI but also a lack of effect, most notably on psychological flexibility as the process of change in ACT.

## 1. Introduction

A large body of research indicates that evidence-based psychological interventions can be effectively translated into self-guided digital mental health interventions (DMHIs) (Ramos et al., 2024). However, significant challenges with adherence and engagement have also been clearly identified as a limitation to the efficacy of DMHIs (Baumel et al., 2019; Lipschitz et al., 2023; Woolley et al., 2024). These adherence issues are even more elevated outside of research contexts (e.g., Woolley et al., 2024), with one study finding that adherence rates were four times lower when DMHIs were delivered as a “real-world” service outside of a controlled study (Baumel et al., 2019). In so far as all of the sessions included in a DMHI are essential to providing the necessary concepts, skills, and intervention dosage, adherence is a significant issue.

One innovative solution to this challenge is to modify DMHIs to reduce the demands of adherence to better match how long many people engage in them, most notably by reducing a DMHI into a single-session intervention (Loades & Schleider, 2023). Although such a brief intervention provides much less training and time for skill acquisition, strengthening, and generalization, it also has the benefit of being intentionally designed so that people who otherwise would dropout after a session receive the most important content. This is not to say a single-session DMHI is an adequate replacement for the broad and diverse mental health needs in a population, but it may provide a key component of a portfolio of resources offered. For example, such a single-session DMHI could be integrated into existing systems, such as a preventative intervention that is less intensive for those not seeking treatment (Cohen et al., 2024). Ideally, a clear treatment model is available to guide the content provided in a single-session DMHI so that it efficiently targets processes of change with specific evidence-based methods.

Acceptance and commitment therapy (ACT) (Hayes et al., 2012) is well suited for such a single session DMHI format. Over 50 randomized controlled trials have found ACT to be efficacious as a DMHI across a wide range of populations (Klimczak et al., 2023). ACT specifies a clear set of treatment components that target psychological flexibility processes, including engaging in values-based actions, being mindfully aware, and being open to internal experiences. Consistent with the theoretical model, ACT DMHIs have been found to improve mental health outcomes through targeting psychological flexibility (e.g., Klimczak et al., 2023; Macri & Rogge, 2024). As a treatment approach defined by its functional impact on psychological flexibility, ACT can be adapted into a flexible range of formats, including as a single-session intervention. For example, several studies have found ACT to be efficacious in a single-session workshop format for individuals with chronic health conditions (Dochat et al., 2021). Although there has been less research on single-session ACT DMHIs, preliminary research suggests it can be efficacious for improving college mental health (Firestone et al., 2019; Henriques Wollach et al., 2020), academic success (Chase et al., 2013), and treatment seeking (Tobias et al., 2022). Further research is needed to determine whether ACT can be effectively delivered in a single-session DMHI format.

The current study sought to conduct an initial pilot evaluation of a single-session ACT DMHI called ACT Guide Lite. This program was designed as a single-session version of a longer ACT DMHI called ACT Guide, which is based on a dismantling trial with university students (Levin et al., 2020) and has since been disseminated as a publicly available service to university students (Davis et al., 2024) and the general public (Woolley et al., 2024). Similar to other DMHIs, we have found significant adherence challenges with ACT Guide, particularly when it is accessed as a publicly available service outside of research, with one analysis finding only 15% of users who purchased the program completed all twelve sessions (Woolley et al., 2024). A single-session version of ACT Guide provides the opportunity to design a “crash course” ACT DMHI that covers key skills and exercises that users might otherwise not receive if they struggle with adherence. Among the variety of potential uses for a single-session ACT DMHI, this could provide a low-intensity preventative resource in non-clinical contexts, such as a university psychology course. Such a program could teach ACT skills found to improve psychological flexibility and mental health in college students (e.g., Levin et al., 2020) in a format that might be particularly acceptable given the ease of access and low burden of participation.

Thus, the current study was a pilot randomized controlled trial comparing ACT Guide Lite to a waitlist condition as a preliminary evaluation of a single-session ACT DMHI. Processes of change and mental health outcomes were assessed at baseline and at 1-week and 1-month follow-ups. Pre-registered hypotheses (NCT06139718) included the following: (1) participants assigned to ACT Guide Lite will improve more on the primary therapeutic process of change, psychological flexibility, relative to those not receiving intervention; (2) participants assigned to ACT Guide Lite will improve more on distress, well-being, and interest in seeking help relative to those not receiving intervention; and (3) ACT Guide Lite will be acceptable to college students as indicated by recruitment rates, rates of completing ACT Guide Lite, and self-reported program satisfaction.

## 2. Method

### 2.1. Participants

A sample of 61 Utah State University (USU) students receiving course credit in a psychology class were recruited for this study. The inclusion criteria were purposefully broad to match the non-clinical, universal prevention context: (1) being 18 years of age or older and (2) a current USU student. Students were excluded if they reported previously using ACT Guide, which was being broadly disseminated as a DMHI to USU students for free at that time. Participants were recruited through the Sona research platform as well as announcements made in classes and flyers.

### 2.2. Procedure

This study was approved by the USU Institutional Review Board and was pre-registered at clinicaltrials.gov (NCT06139718). All study procedures were completed online through the Qualtrics survey platform. After providing online informed consent, the participants completed an online baseline survey and were then automatically randomized (1:1 allocation ratio) to either ACT or a waitlist condition. Randomization was set in blocks of 10 to help ensure equal sample sizes in each condition over time.

The participants assigned to ACT were given a link to complete the ACT Guide Lite program. The participants could complete the single-session program right after randomization or choose to wait to complete it. An email prompt was sent from the research team if the program was not completed within a few days. The participants assigned to the waitlist condition were asked to complete the other study assessments and were provided access to the program after the final assessment point.

A post-intervention assessment was included at the end of the ACT Guide Lite program just for participants in the ACT condition, which assessed program satisfaction variables. Follow-up assessment surveys were sent to participants in both conditions at one-week and one-month follow-up time points.

### 2.3. ACT Guide Lite

ACT Guide Lite is a fully automated, asynchronous digital intervention, providing a consistent delivery of ACT skills over time and across users in a convenient to access format. The program consists of key skills from the larger 12-session ACT Guide program (Davis et al., 2024). ACT skills were selected to target a range of psychological flexibility subprocesses (as opposed to some single-session DMHIs that target just a single subprocess such as values. Firestone et al. (2019) ACT Guide Lite was iteratively refined through think aloud testing with users from both clinical and non-clinical samples (*n* = 12). This process facilitated the identification of usability and acceptability factors that needed to be addressed prior to launching the program (e.g., modifying examples to be more relatable, automated option to email the user their session summary, adding audio-guided option to the acceptance exercise).

ACT Guide Lite was divided into a series of sections. Pan = 2 rt 1 (explore what matters) started with an orientation page, followed by a series of values exercises: (1) reflecting on the importance of and your success in different domains of life, (2) selecting a personally meaningful value to work on, and (3) a writing exercise to reflect on what this value means to you. Part 2 (learn a new way) targeted acceptance and cognitive defusion through (1) identifying challenging thoughts and feelings; (2) completing an interactive passengers on the bus metaphor to explore how fused and avoidant responses lead to getting more stuck and driving with one’s passengers as an accepting, defused alternative; (3) identifying examples of “driving with passengers” and an action they could take with difficult passengers; and (4) how to engage in “driving with passengers” in a compassionate way. Part 3 (practice a new skill) introduced the participants to a series of steps to practice being aware and open to difficult feelings when they arise (acknowledge it, observe how it feels in your body, breathe into it, allow it to be there, and then expand your awareness). This included (1) examples of practicing the skill, (2) identifying a feeling that is present to practice with (or connecting with a feeling to bring into a practice session), and (3) practicing the series of exercise steps following either an audio guided recording or an interactive series of text-based pages. Part 4 (commit to a goal) then guided the participants to commit to a values-based goal to work on while practicing acceptance and defusion. This included (1) identifying a feasible goal for this week, (2) writing about how this goal is connected to one’s values, (3) identifying “passengers” that might come up, (4) selecting strategies to use to help practice ACT skills with thoughts and feelings that arise while working on this goal, and (5) setting a reminder to work on the goal. The section also included strategies to continue to build on what they learned, including ways to continue to practice ACT skills and other mental health resources they might follow up with (including university counseling services). Finally, a summary was provided to users, including key points from the program and data they entered in exercises, which could be printed or emailed to oneself.

### 2.4. Measures

Comprehensive assessment of acceptance and commitment therapy processes (CompACT) (Francis et al., 2016). The CompACT is a 23-item, self-report measure of psychological flexibility, the primary process of change in ACT. Items (e.g., “I can identify the things that really matter to me in life and pursue them”) are rated on a 7-point scale ranging from 0 (strongly disagree) to 6 (strongly agree). Total scores range from 0 to 138, with higher scores indicating greater psychological flexibility. The CompACT demonstrated good internal consistency across all time points (*α* = 0.84–0.89) in the present study.

Mental Health Continuum-Short Form (MHC-SF) (Keyes, 2005). The MHC-SF is a 14-item, self-report measure of emotional, social, and psychological well-being. Items are rated on a 6-point Likert scale ranging from 0 (never) to 5 (everyday), with total scores ranging from 0 to 70; higher scores indicate greater well-being. The MHC-SF demonstrated excellent internal across all time points (*α* = 0.92–0.94) in the present study.

Depression Anxiety Stress Scale (DASS) (Lovibond & Lovibond, 1995). The 21-item DASS is a self-report measure of psychological distress, with subscales assessing depression, anxiety, and stress. Items are rated on a 4-point Likert scale from 0 (never) to 3 (almost always). The total score on the DASS ranges from 0 to 120, with higher scores indicating higher distress. The DASS total score demonstrated good to excellent internal consistency across all time points (*α* = 0.84–0.91).

General Help Seeking Questionnaire (GHSQ) (Wilson et al., 2005). The GHSQ assesses intentions to seek help for a mental health concern from sources including professional and informal supports. The GHSQ has been adapted to include responses assessing self-guided treatment resources (Levin et al., 2017). This adapted version of the GHSQ was used in the present study, with six items assessing help-seeking intentions from a family member, friend, psychiatrist, mental health professional, self-help application, and not help seeking from anyone. Each item is rated on a 7-point scale ranging from 1 (extremely unlikely) to 7 (extremely likely); higher scores indicate greater intent to seek help for a mental health concern if one was present. Items are scored individually, so no internal consistencies were calculated for the GHSQ.

Program satisfaction. At post-intervention, the participants completed a series of program satisfaction items that were adapted from previous DMHI trials (e.g., Levin et al., 2020). The participants in the intervention condition completed eight items assessing aspects of the online program, such as overall satisfaction, perceived quality, helpfulness, and appropriateness of program length. Items are rated on a 6-point Likert scale, ranging from 1 (strongly disagree) to 6 (strongly agree), with higher scores indicating greater satisfaction with the program.

### 2.5. Data Analytic Plan

Program acceptability was examined through descriptive statistics characterizing the number and time it took to recruit participants in the study, the adherence rate of completing ACT Guide Lite in the ACT condition, and self-reported program satisfaction ratings from participants in the ACT condition.

Multilevel models (MLM) with the full intent-to-treat sample were used to test time by condition interactions (i.e., whether participants assigned to ACT intervention improved more over time relative to waitlist) for each outcome, including psychological flexibility (CompACT), well-being (MHC-SF), psychological distress (DASS-21), and intentions to seek help for a mental health concern (GHSQ). To assess statistical assumptions for each outcome, residual diagnostics were examined to assess the normality of residuals and identify potential outliers. Homoscedasticity was evaluated by plotting residuals against fitted values. Independence of errors was assumed within levels, as participants’ measurements were nested over time. No significant violations of these assumptions were observed. For all MLMs, traditional model fit indices (e.g., AIC and BIC) were compared across null (random intercept for each participant), fixed effects (time and condition), and interaction effects (time by condition) to evaluate model fit. Restricted maximum likelihood (REML) criteria were used for final model estimation, and *p*-values were determined based on Satterhwaite’s method. All MLM analyses were conducted in R Studio (R Core Team, 2024) with the lme4 package (Bates et al., 2015).

## 3. Results

### 3.1. Participant Flow and Demographics

**Figure 1:**
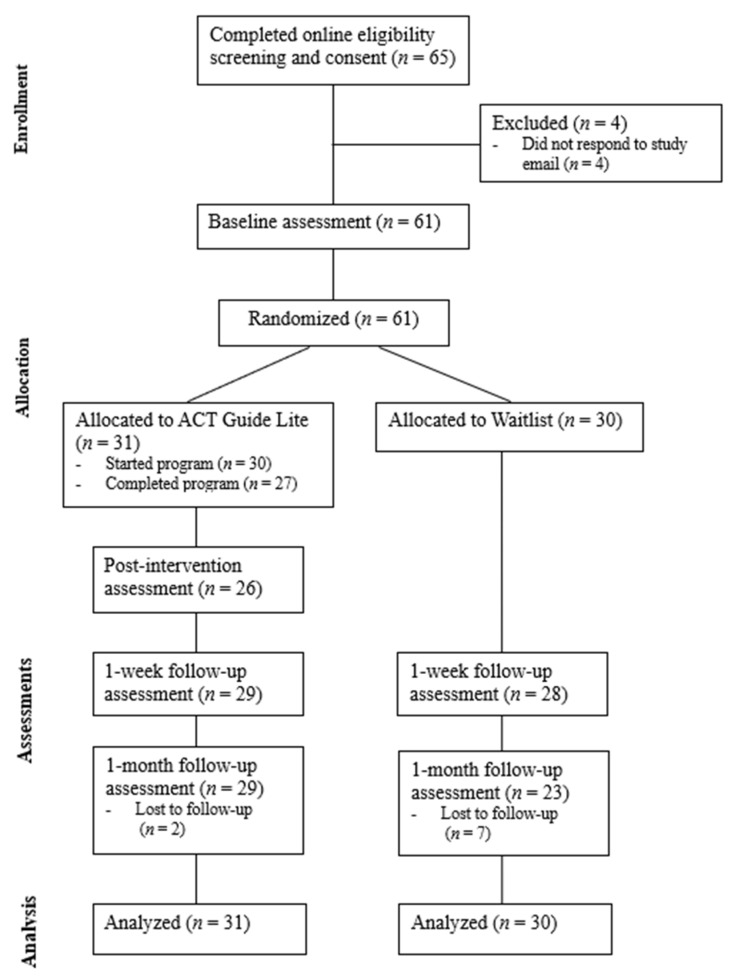
CONSORT diagram for participant flow.

From January to March 2024, 61 participants enrolled in this study (see Figure 1 for participant flow). This was below the pre-specified acceptability target of enrolling 100 participants and represented a small portion of the approximate 1000 students eligible for psychology course credit for participating. Assessment retention rates were relatively high, with 93% completing the 1-week follow-up and 85% completing the 1-month follow-up. To evaluate whether the participants who dropped out differed systematically from those who completed this study, baseline demographic and psychological characteristics (e.g., age, gender, motivation, psychological flexibility, well-being, distress) were compared between groups using Welch’s two-sample *t*-tests for continuous variables and chi-squared analyses for categorical variables. No significant differences were found between the participants who completed follow-up assessments and those who did not (*p* > 0.05).

The participants primarily self-identified as White (*n* = 57, 93%), heterosexual (*n* = 57, 93%), and women (*n* = 48, 79%), with an average age of 21.7 years. Approximately half of the sample (*n* = 31, 51%) were first year undergraduate students. The majority endorsed participating in this study for course credit (*n* = 58, 95%) as opposed to participating to improve one’s mental health (*n* = 2) or for credit and to improve mental health (*n* = 1). On average, the participants endorsed between “slightly disagree” and “slightly agree” in response to “I am motivated to use an online self-help program to improve my mental health and well-being” in both ACT (*M* = 3.42. *SD* = 1.03) and waitlist (*M* = 3.50, *SD* = 0.90) conditions. See Table 1 for all baseline sociodemographic characteristics by condition. No significant differences were found when testing for baseline equivalency across sociodemographic and baseline outcome variables (*p* > 0.05); see Table 2 for outcome scores across all time points by condition.

### 3.2. Program Acceptability

Out of the 31 participants randomized to ACT, 27 participants completed the single-session program (87.1% completion rate), indicating high program acceptability in terms of adherence. An exploratory analysis examined the time the participants spent to complete the intervention. After removing three outliers (47.70, 47.52, and 2.04 h), the median duration for the participants to complete the program was 32.86 min (*M* = 40.20, *SD* = 23.11, range = 14.07 to 96.75 min).

The participants who completed ACT Guide Lite provided program satisfaction ratings at the end of the intervention (see Table 3). The participants provided high program satisfaction ratings averaging between 5 (“agree”) and the maximum score of 6 (“strongly agree”) on items including overall satisfaction, perceived helpfulness, ease of use, receiving actionable skills, and that it would be helpful for others. The only satisfaction item that was rated low was whether ACT Guide Lite made participants more interested in therapy, with the average being near 4 (“slightly agree”) and only 69.2% rating a 4 or higher (consistent with the lack of effects on the GHSQ reported in the next section).

In terms of program length, at post-intervention, the participants agreed (*M* = 5.12) that the program was long enough to meet their goals and disagreed on average (*M* = 2.73) that the program would be better if it was shorter. However, the participants were more divided at 1-week follow-up on the question “I wish ACT Guide Lite had more sessions that I could continue to complete each week” (*M* = 3.86, *SD* = 1.27, range = 1–6), suggesting mixed opinions about the program only being a single session over time.

### 3.3. Intervention Outcomes

The only significant difference between conditions over time was found for well-being (MHC-SF), as seen in Table 4. The time by condition interaction for MHC-SF (*β* = 1.05, *p* = 0.037) indicates the participants assigned to ACT improved on well-being scores over time compared to the waitlist control, with a small between-condition effect from baseline to 1-month follow-up (*d* = 0.29). Both psychological flexibility (CompACT) and distress (DASS-21) models had a significant fixed effect of time, indicating scores improved over time regardless of condition, but there was no significant time by condition interaction. The baseline to follow-up between-condition effect sizes were very small for the CompACT (*d* = 0.18) and DASS (*d* = −0.05), suggesting non-significant time by condition effects were not due to limited statistical power. The best-fitting model for all mental health help seeking (GHSQ) items was the null model, indicating that time and condition did not meaningfully explain the variability in help seeking behavior beyond what was captured by the random effect of individual participants.

## 4. Discussion

This pilot trial sought to examine the acceptability and preliminary efficacy of a single-session ACT DMHI with college students. Most participants completed the program and rated it high on self-reported satisfaction questions, suggesting high acceptability, although recruitment was below the target enrollment. Mixed effects were found for intervention outcomes, with small improvements in the ACT condition relative to waitlist on well-being, but no differences were found between conditions on the primary outcome of psychological flexibility, measures of distress, or interest in help seeking. Overall, these results provide mixed support for the potential acceptability and efficacy of a single-session ACT DMHI.

### 4.1. Acceptability

This study sought to evaluate one way a single-session ACT DMHI might be deployed, which is as a low-intensity mental health resource that is integrated into a classroom setting. ACT is a promising approach for preventing mental health concerns by targeting psychological flexibility as a common core process (Biglan et al., 2008), but work is needed in adapting ACT to the needs of a prevention context. For example, past research providing a multi-session ACT DMHI to prevent mental health problems among college students was found to have low acceptability due in part to the effort required for adherence (Levin et al., 2016). A single-session design might provide the benefit of being flexible and low intensity enough for non-clinical settings such that they can be incorporated into existing systems such as a course (Cohen et al., 2024) and are brief enough that someone who is not actively seeking help may be willing to complete it. We did find some support for the acceptability of this approach. Even though the participants were generally not interested in completing a DMHI to improve their mental health and participated for course credit, adherence rates and program satisfaction ratings were still high. However, most students enrolled in eligible psychology courses during the semester (approximately 1000) did not participate in this study. Providing mental health interventions in classes is a common method for universal prevention programs in universities (Conley et al., 2015), but these results suggest our specific approach of offering ACT as a single-session intervention for course credit might not attract a large portion of students in a non-clinical context. This potential limited interest in a single-session intervention matches the findings from a naturalistic study of a public version of the ACT Guide Lite program, in which we found the vast majority of college students chose to enroll in a long, multi-session ACT DMHI rather than the single-session ACT Guide Lite program (Bowers et al., n.d.). Although many students might struggle to complete a multi-session program, it appears that offering a single-session program might not lead to the high enrollment needed for successful universal prevention programs.

### 4.2. Preliminary Efficacy

Consistent with the goals of ACT in improving values-based living and with the non-clinical focus of this study, the results indicated that ACT Guide Lite might lead to improvements in positive mental health. Prior research has similarly found that ACT is efficacious in improving well-being among college students (Howell & Passmore, 2019). These findings suggest a single-session ACT DMHI could be used to promote positive mental health among students, an important outcome above and beyond preventing or reducing psychological distress and disorders (Keyes, 2005).

However, the lack of between-condition effects on the other outcomes raises questions regarding the impact of the intervention. The lack of effect on psychological distress may be due in part to the non-clinical sample, given a prior study with a brief ACT DMHI found that depression and anxiety symptoms only improved in the subgroup of students with elevated symptoms (Levin et al., 2014). ACT Guide Lite also failed to improve intentions to seek help for a mental health condition, despite including content focused on help seeking, which raises questions regarding its use as an initial treatment step to increase interest in seeking more intensive services. This contrasts with a prior study that found a single-session ACT DMHI increased treatment-seeking intentions (Tobias et al., 2022).

The more significant concern is the lack of intervention effects on psychological flexibility, which is the primary target in ACT. A large body of prior research indicates psychological flexibility mediates the effects of ACT for a wide range of mental health conditions (Klimczak et al., 2023; Macri & Rogge, 2024). Theoretically, when ACT fails to improve psychological flexibility, it would suggest a failure of the intervention to effectively deliver ACT in terms of its functional impact, and thus, a lack of other mental health improvements would be expected. This suggests the single-session ACT DMHI failed to effectively deliver ACT, but this conflicts with the improvements in well-being. Potential explanations for these mixed results include that the improvements in well-being are due to more general effects of an intervention (particularly against a no-treatment control) or that the psychological flexibility process measure was not sensitive to detecting the effects of the ACT DMHI on this process of change. There is some potential for this latter point given research indicating that mediation findings for psychological flexibility with ACT are weaker in student samples (Macri & Rogge, 2024), that effects of ACT on psychological flexibility among college students may only be significant in clinical samples (Hsu et al., 2023), and that more context-specific psychological flexibility measures are better at detecting the effects of ACT (Ong et al., 2019). This study did have a relatively small sample due to lower than expected enrollment rates, but this seems to be a less likely explanation for non-significant effects given the effect size for pre- to 1-month follow-up between ACT and waitlist was below the cutoff for a small effect for psychological flexibility (*d* = 0.18) and distress (*d* = −0.05), while a significant small effect size was found for positive mental health (*d* = 0.29). We decided to not extend recruitment beyond the planned time window given the very small effect sizes for other outcomes and that results clearly indicated mixed findings for the intervention (including low sign-up rates) when delivered in this context with the obtained data, which are important to report. It also seems unlikely that the lack of intervention effects was due to the assessment time window of 1-month missing delayed improvements that take more time to be observable given the single-session intervention is focused on more immediate effects on outcomes, particularly the targeted process of change.

Several past studies have similarly found mixed or null results on psychological flexibility and mental health outcomes when evaluating ACT in a non-clinical, school-based context with college students or adolescents, either through in-person (e.g., Burckhardt et al., 2017; Van der Gucht et al., 2016) or DMHI formats (e.g., Lappalainen et al., 2021; Larsson et al., 2022; Levin et al., 2016; Morey et al., 2024). For example, a large cluster-randomized trial that compared an ACT prevention program delivered by a teacher to usual curriculum in a sample of 586 adolescents found no between-condition effects on psychological flexibility or mental health outcomes (Van der Gucht et al., 2016). Thus, this study adds to a body of research that runs counter to the larger positive evidence base of ACT, highlighting challenges in moving psychological flexibility and resulting mental health outcomes with ACT interventions in a non-clinical, school-based context. This is also consistent with the broader literature indicating mixed findings for universal interventions in school settings to address mental health in the full population of students (Greenberg & Abenavoli, 2016), although meta-analyses indicate that at least some forms of skill-focused universal prevention programs are efficacious in college samples (Conley et al., 2015).

Given the mixed findings for ACT in a non-clinical, school-based prevention context, it is unclear whether the lack of effects on psychological flexibility is due to a limitation in the ACT Guide Lite single-session DMHI or in applying ACT in this context more broadly. Future research would benefit from evaluating ACT Guide Lite in other targeted contexts and clinical populations. The laboratory-based component research on ACT has involved testing brief single-session ACT interventions, often delivered in a digital self-guided format. These component studies have consistently found positive results for ACT components in isolation and combination in digital single-session formats (Levin et al., 2012). However, these studies typically are much more focused on proximal measures of psychological flexibility and mental health outcomes, with the intervention heavily tailored to moving the targeted measure. This is similar in the broader single-session intervention literature, where there is often a more targeted focus of the intervention and associated measures (Schleider et al., 2025). For example, prior single-session ACT DMHI trials in college student samples have been more targeted interventions with a subset of ACT components for specific outcomes, including the effects of values and goal setting on academic success (Chase et al., 2013), values on values-consistent living (Firestone et al., 2019), and cognitive defusion on the believability and discomfort from negative self-critical thoughts (Henriques Wollach et al., 2020). In contrast, this study tested a single-session program designed to incorporate multiple components of ACT that target distinct psychological flexibility subprocesses and applied in a general mental health context that could be used transdiagnostically. Similarly, the measures selected were broad measures of mental health and psychological flexibility. These general features of the intervention and assessment method may have reduced sensitivity to detecting treatment effects. That said, it is also an empirical question as to whether a single-session ACT DMHI is more effective if targeting a single ACT component (e.g., values, cognitive defusion) versus combining several components, which might be tested in future research.

### 4.3. Limitations

This study was limited by only comparing the intervention to a waitlist control. It is unclear whether the effects that were found between conditions were due to the active ACT components of the DMHI or to other factors, such as placebo and demand characteristics common to receiving any intervention in a study context. A waitlist control can provide useful preliminary information on a signal for potential efficacy, but it fails to answer more useful questions that could move the field further forward. Future research is needed to answer the core question underlying a single-session approach—“how much intervention is needed for ACT to be efficacious?” This might be better answered with a study that randomizes participants to either a single-session DMHI or versions with more sessions, determining if effects are equivalent with this shorter length or for whom such brief interventions are sufficient. Researchers have begun to conduct such research comparing the length of therapist-delivered ACT protocols (Kroska et al., 2020; Pedersen et al., 2019). Such work is critically needed with evaluating ACT DMHIs given the challenges with adherence (Baumel et al., 2019; Lipschitz et al., 2023; Woolley et al., 2024) and potential benefits if single-session or other briefer interventions are equally effective for some individuals.

Although this study found high adherence rates, it is unclear the degree to which this indicated actual engagement in the intervention (e.g., how carefully they attended to the content and whether they fully participated in exercises and learned relevant skills). Data on time spent in the program indicated none of the participants quickly skipped through, with the fastest completer still taking 14 min. We have found in prior research that a longer duration in sessions correlates with greater improvements in psychological flexibility processes (Levin et al., 2016). However, there were also notable outliers for a long duration in the program, which demonstrates the limitations in this time data and the potential it is also capturing the degree to which participants were engaged in other activities while leaving the program open. Given the multifaceted nature of DMHI engagement (Perski et al., 2017), future research would benefit from assessing other variables, including, importantly, what participants actually learn from the program (Käll & Andersson, 2023).

Although this study purposefully sought to pilot ACT Guide Lite as a prevention program for use in a non-clinical context, this also limited the ability to determine the potential efficacy of the single-session ACT DMHI, particularly given the mixed results for this universal prevention approach with ACT in the broader literature (e.g., Burckhardt et al., 2017; Lappalainen et al., 2021; Larsson et al., 2022; Levin et al., 2016; Van der Gucht et al., 2016) and universal intervention programs more broadly (Conley et al., 2015; Greenberg & Abenavoli, 2016). The participants openly acknowledged they participated for course credit rather than to improve their mental health, highlighting that the incentives used might have affected the impact of the intervention. The lack of a distress cutoff might have also led to floor effects weakening the sensitivity to detecting the impact of ACT Guide Lite on distress, which has been found in prior ACT DMHI prevention studies with college students (e.g., Levin et al., 2014). Further, the small sample size resulting from recruitment challenges reduces the reliability and potential replicability of the results, although the sample was sufficiently large within a MLM approach to detect a small between-condition effect. Small effects are expected from universal prevention programs given the heterogeneity of the sample and their potential response to the intervention, but recruiting a much larger sample would have allowed for a comprehensive assessment of the benefits of the intervention such as subgroup effects (Greenberg & Abenavoli, 2016). In addition, the small sample size reduces the reliability of the results given findings may change with a larger sample that is less affected by potential outliers and unique sample characteristics. Finally, the high rate of women and White participants limits the generalizability of these findings to more diverse and minoritized student populations.

## 5. Conclusions

Overall, this pilot study found mixed support for a single-session ACT DMHI delivered in a non-clinical context for college students. Positive findings included high adherence and satisfaction ratings as well as small significant effects on well-being relative to no intervention. Negative findings included lower than anticipated enrollment rates and no between-condition effects on psychological flexibility, distress, or help seeking intentions. These results may be explained by the challenges and limitations in delivering ACT as a school-based prevention program and adds to the literature highlighting these issues. Single-session ACT DMHIs hold significant promise in providing a low-intensity resource that can address service gaps for individuals who might struggle with adhering to more intensive interventions. Future research would benefit from testing this single-session ACT DMHI in targeted clinical populations and with active control conditions, including comparing effects with multi-session ACT programs and targeting isolated versus combined ACT treatment components.

## Figures and Tables

**Table 1:** Baseline sociodemographic characteristics.

Characteristic	ACT (*n* = 31)	Waitlist (*n* = 30)
Age *M* (*SD*)	22.58 (8.41)	20.83 (2.68)
Gender *n* (%)		
Man	8 (26%)	5 (17%)
Woman	23 (74%)	25 (83%)
Race ^1^ *n* (%)		
Asian	0 (0%)	1 (3%)
Hispanic, Latino, or Spanish Origin	1 (3%)	2 (7%)
White or European American	30 (97%)	29 (97%)
Sexual Orientation *n* (%)		
Asexual	1 (3%)	0 (0%)
Bisexual	1 (3%)	0 (0%)
Heterosexual or straight	28 (90%)	29 (97%)
Pansexual	1 (3%)	0 (0%)
Queer	0 (0%)	1 (3%)
School Year *n* (%)		
First year (Freshman)	15 (48%)	16 (53%)
Second year (Sophomore)	10 (32%)	8 (27%)
Third year (Junior)	2 (6%)	1 (3%)
Fourth year (Senior)	3 (10%)	3 (10%)
Fifth year or higher	1 (3%)	2 (7%)
Study Participation Reason *n* (%)		
For course credit/extra credit	30 (97%)	28 (93%)
For help improving my mental health and well-being	1 (3%)	1 (3%)
Both for course credit and improving well-being	0 (0%)	1 (3%)
Baseline Concomitant Treatment *n* (%)		
Psychotherapy	5 (16%)	8 (27%)
Psychotropic Medication	7 (23%)	8 (27%)
Motivation *n* (%)		
2 (disagree)	2 (6%)	1 (3%)
3 (slightly disagree)	2 (6%)	3 (10%)
4 (slightly agree)	12 (39%)	8 (27%)
5 (agree)	11 (35%)	16 (53%)
6 (strongly agree)	4 (13%)	2 (7%)

^1^ Categories not mutually exclusive.

**Table 2:** Averages and standard deviations for all outcome measures and time points separated by condition.

Outcome Measures	ACT			Waitlist		
	Baseline (*n* = 31)	1-Week Follow-Up (*n* = 29)	1-Month Follow-Up (*n* = 29)	Baseline (*n* = 30)	1-Week Follow-Up (*n* = 28)	1-Month Follow-Up (*n* = 23)
CompACT	80.4 (18.6)	83.9 (15.7)	86.7 (18.9)	85.2 (14.8)	86.2 (14.1)	88.5 (12.6)
MHC-SF	44.0 (16.0)	44.2 (13.6)	48.2 (13.9)	50.2 (11.4)	49.8 (9.4)	50.3 (11.3)
DASS-21	36.3 (24.6)	30.4 (16.4)	27.7 (18.9)	32.0 (19.0)	26.4 (14.3)	24.5 (14.5)
GHSQ						
Family member	5.0 (2.1)	5.6 (1.6)	5.1 (1.8)	5.8 (1.7)	5.9 (1.5)	6.1 (1.1)
Friend	5.0 (1.7)	4.9 (1.9)	5.1 (2.1)	5.1 (1.7)	5.2 (1.6)	5.2 (1.4)
Psychiatrist	3.2 (1.8)	3.0 (1.7)	3.1 (1.9)	3.5 (1.7)	3.5 (1.6)	4.2 (1.8)
Mental health professional	3.9 (1.9)	3.6 (1.9)	3.9 (2.0)	4.5 (1.9)	4.5 (2.0)	4.9 (2.1)
Self-help app/website	3.3 (1.7)	3.2 (1.3)	3.8 (2.0)	3.3 (2.0)	3.0 (1.7)	3.5 (2.0)
Not seek help from anyone	2.6 (1.9)	2.9 (2.0)	3.0 (2.2)	2.3 (1.5)	2.2 (1.6)	2.1 (1.6)

**Table 3:** Program satisfaction ratings for participants in the online intervention condition.

Satisfaction Item	Mean (SD)	% Rating ≥ 4 “Slightly Agree”
Overall, I was satisfied with the quality of ACT Guide Lite	5.23 (1.07)	92.31%
ACT Guide Lite was helpful for me	4.92 (1.20)	92.31%
ACT Guide Lite was easy to use	5.58 (0.76)	96.15%
ACT Guide Lite gave me actionable skills to work on my mental health	5.19 (0.94)	96.15%
ACT Guide Lite made me more interested in and willing to go to therapy	4.04 (1.31)	69.23%
I think ACT Guide Lite would be helpful for others struggling with mental health problems	5.31 (0.79)	96.15%
ACT Guide Lite was long enough and included enough information to meet my goals for using the program	5.12 (0.86)	96.15%
ACT Guide Lite would be better if it was shorter and had fewer skills/concepts *	2.73 (1.15)	19.23%

Note. Satisfaction items were rated on a 1 (strongly disagree) to 6 (strongly agree) scale, with 4 indicating “slightly agree”. * indicates a reverse scored item such that disagreeing (3 or lower) indicates greater satisfaction with the current program.

**Table 4:** Estimated marginal means and 95% confidence intervals from multilevel models of primary outcomes.

Parameter	CompACT	MHC-SF Total	DASS-21 Total
Intercept	84.44 [78.81; 90.07] *	49.55 [44.97; 54.13] *	30.97 [24.87; 37.08] *
Weeks	0.99 [0.32; 1.66] *	−0.02 [−0.75; 0.70]	−1.50 [−2.55; −0.44] *
Condition (ACT)	−1.84 [−9.62; 5.93]	−5.64 [−12.06; 0.78]	3.22 [−5.05; 11.49]
Weeks × ACT		1.05 [0.08; 2.02] *	
AIC	1310.81	1214.73	1417.11
BIC	1326.49	1233.54	1432.79
Log Likelihood	−650.41	−601.36	−703.56
Number of observations	170	170	170
Number of participants	61	61	61

* Null hypothesis outside the confidence interval.

## Data Availability

De-identified data from this study are not available in a public archive. De-identified data from this study will be made available (as allowable according to institutional IRB standards) by emailing the corresponding author.
